# The Triglyceride Paradox in Stroke Survivors: A Prospective Study

**DOI:** 10.1155/2013/870608

**Published:** 2013-02-25

**Authors:** Minal Jain, Anunaya Jain, Neeraja Yerragondu, Robert D. Brown, Alejandro Rabinstein, Babak S. Jahromi, Lekshmi Vaidyanathan, Brian Blyth, Latha Ganti Stead

**Affiliations:** ^1^Department of Neurosurgery, University of Rochester Medical Center, Rochester, NY 14627, USA; ^2^Department of Emergency Medicine, Mayo Clinic College of Medicine, Rochester, MN 55905, USA; ^3^Department of Neurology, Mayo Clinic, Rochester, MN 55905, USA; ^4^Department of Emergency Medicine, University of Rochester Medical Center, Rochester, NY 14627, USA; ^5^Departments of Emergency Medicine and Neurological Surgery, University of Florida College of Medicine, Gainesville, FL 32608, USA

## Abstract

*Objective*. The purpose of our study was to understand the association between serum triglycerides and outcomes in acute ischemic stroke (AIS) patients. *Methods*. A cohort of all adult patients presenting to the Emergency Department (ED) with an AIS from March 2004 to December 2005 were selected. The lipid profile levels were measured within 24 hours of stroke onset. Demographics, admission stroke severity (NIHSS), functional outcome at discharge (modified Rankin Scale (mRS)), and mortality at 3 months were recorded. *Results*. The final cohort consisted of 334 subjects. A lower level of triglycerides at presentation was found to be significantly associated with worse National Institutes of Health Stroke Scale (NIHSS) (*P* = 0.004), worse mRS (*P* = 0.02), and death at 3 months (*P* = 0.0035). After adjusting for age and gender and NIHSS, the association between triglyceride and mortality at 3 months was not significant (*P* = 0.26). *Conclusion*. Lower triglyceride levels seem to be associated with a worse prognosis in AIS.

## 1. Introduction

Ischemic stroke is the third leading cause of death worldwide with 5 million annual deaths [1]. Every years approximately 795,000 people experience a stroke and someone dies of stroke in the United States every 3-4 minutes [2]. One of the prominent objectives of stroke research has been to investigate factors that could potentially improve stroke outcome. Acute factors affecting stroke outcome such as awareness of symptoms [3], blood pressure [4], elevated glucose [5], and electrocardiographic intervals [6] have all been of interest. In this paper, we turn our attention to the lipid profile in the setting of acute ischemic stroke. 

It is known that high serum lipids including triglycerides constitute major risk factors for stroke [7, 8]. Little has been reported to date on the role of triglycerides in acute stroke and their role in poststroke recovery. The few studies that have evaluated this association have reported diverse associations [9–12]. The purpose of our research was to study the relation between poststroke serum triglyceride levels and functional stroke outcome, in a large prospective cohort of acute ischemic stroke patients.

## 2. Methods

This study is a substudy of an Emergency Department (ED) acute stroke registry. The ED Stroke registry is an institutional review board approved prospective consecutive cohort study. All adult patients presenting to the ED of our academic tertiary care centre with a diagnosis of acute ischemic stroke were eligible for inclusion. For this substudy, pediatric stroke patients (age <18 years), patients with hemorrhagic stroke and transient ischemic attack were excluded. Further, adult stroke patients who did not have a lipid profile measured within 24 hours of admission to the hospital were also excluded. This study covers a 22-month period from March 2004 to December 2005. All patients were consented on presentation, and their medical records were reviewed.

Stroke severity at the time of admission was determined using the NIH stroke scale (NIHSS). The functional disability on discharge was determined from the modified Rankin scale (mRS). Patients with an mRS ≥3 were considered to have a poor outcome. In the past, researchers have used dichotomized modified Rankin scores, similar to ours, to study functional outcome after stroke [13]. 

As a part of the stroke management protocol, fasting blood samples were collected for lipid profile analysis. Blood was collected in plain tubes and centrifuged, and 1 mL of serum was introduced into the analyzer for lipid analysis. The analyzer measured the serum cholesterol, High Density Lipoprotein (HDL), and triglyceride levels using the enzymatic colorimetric method and calculated the serum Low Density Lipoprotein (LDL) using the Friedewald equation [14]. 

All patients were prospectively followed up for 3 months from the time of stroke for mortality outcomes. Data analysis was performed with JMP 8.0. The Kruskal-Wilcoxon test was used for nonparametric analysis. All associations between discrete variables were determined using the Pearson/Fisher's test where applicable. Triglyceride level was considered the dependent variable with mortality and functional outcome being the independent variables. Logistic Regression analysis was performed to evaluate the association between triglyceride and poor outcome after controlling for potential confounders. Cox Proportional Hazard Analysis was done to evaluate if triglyceride was an independent predictor of death at 3 months. The level of significance of association was predetermined at *P* < 0.05 for all analyses. 

## 3. Results

All 510 patients presenting with stroke during the study period were consented; of these 334 had initial lipid profile measures and, hence, were included. 

### 3.1. General Characteristics (Table 1)

The median age for the cohort was 74 years, (interquartile range (IQR) 62–81.25 years). Females comprised 44.6% of the cohort. The median age for males was 72 years (IQR 60–81 years) compared to the 75-year median age for females (IQR 64–83.5 years). 10% of the female subset had age <50 years. There was no significant age difference between the genders (*P* = 0.052). Nearly 34% of subjects in the cohort were on prior treatment with statins (HMG-coA reductase inhibitors), at the time of enrollment.

### 3.2. Lipid Profile Analysis

The lipid profile for patients within the first 24 hours of presentation is shown in Table 2. We found that older patients tended to have lower cholesterol levels (*P* = 0.0054). Similar inverse linear associations were also found between total triglyceride and age (*P* = 0.0180) and LDL and age (*P* = 0.0011). No significant association was observed between HDL concentrations and age (*P* = 0.0830). We found females to have higher cholesterol (median 4.73 mmol/L, IQR 4.1–5.41 mmol/L) as compared to males (median 4.45 mmol/L, IQR 3.62–5.26 mmol/L; *P* = 0.024). Females also had higher HDL cholesterol (median 1.4 mmol/L, IQR 1.18–1.69 mmol/L) when compared to males (median 1.16 mmol/L, IQR 0.98–1.42; *P* < 0.001). There were no statistically significant relationships between LDL and gender (*P* = 0.1823) or triglycerides and gender (*P* = 0.6992). 

A bivariate analysis of triglycerides and NIHSS revealed a statistically significant inverse linear relationship between the two (*P* = 0.0015) revealing that high triglycerides were associated with low NIHSS scores. However, we did not find any associations between NIHSS and cholesterol (*P* = 0.4575), HDL (*P* = 0.1237), and LDL (*P* = 0.1422) levels. There was also no significant association between NIHSS and age (*P* = 0.24), gender (*P* = 0.41), and prior statin use (*P* = 0.08), respectively. The Pearson's correlation coefficients for age and NIHSS with respect to lipid levels are reported in Table 3. Patients using statins prior to stroke had lower total cholesterol (median 4.03 mmol/L, IQR 3.44–5.02 mmol/L versus median 4.78 mmol/L, IQR 4.06–5.46 mmol/L, *P* < 0.0001), lower LDL (median 2.09 mmol/L, IQR 1.68–2.65 mmol/L versus median 2.72, IQR 2.09–3.39 mmol/L, *P* < 0.0001) and lower HDL (median 1.16 mmol/L, IQR 0.96–1.4 mmol/L versus median 1.34 mmol/L, IQR 1.11–1.63 mmol/L, *P* = 0.0002), but higher triglycerides levels (median 1.51 mmol/L, IQR 1.09–2.21 mmol/L versus median 1.28 mmol/L, IQR 0.91–1.76 mmol/L, *P* = 0.0056). The triglyceride values were, however, within the desirable normal range (2.26 mmol/L). The lipid profile across both gender and statin is shown in Table 3.

### 3.3. Functional Outcome Analysis: mRS

The median mRS at discharge was 3, IQR 2–4. A significant proportion (65.2%) had poor outcome (mRS ≥3). There was a positive linear relationship between age and mRS at discharge (Pearson's correlation coefficient 0.2631, 95% CI 0.16–0.36, *P* ≤ 0.0001). Males tended to have significantly better outcome as compared to females (mean mRS 2.8 (95% CI 2.6–3) versus mean mRS 3.3 95% CI (3.1–3.5); *P* = 0.0026). 

A bivariate analysis of all individual lipid levels and outcome at discharge revealed a statistically significant relation only between low triglyceride levels and high mRS at discharge (*P* = 0.0197). However, the association for all other lipids-cholesterol, HDL and LDL and outcome at discharge was not statistically significant. The Pearson's correlation between the mRS at discharge and lipid levels are shown in Table 3. 

There was no significant association between patients who were on prior treatment with statin and mRS at discharge (*P* = −0.2349). 

### 3.4. Outcome Analysis: Death within 3 Months

At three months after stroke, 9.6% were reported dead. Patients, who died within this follow-up time frame, were older (median age 80.5 years; IQR 66–86.75 years) than survivors (median age 73 years, IQR 61.75–81 years; *P* = 0.0124). There was no significant association between gender and death within 3 months (*P* = 0.9180). As expected, patients who died within 3 months had significantly worse strokes, as measured on the NIHSS (median score 16, IQR 12–21, v/s median score 4, IQR 2–8; *P* < 0.0001). 

 Subjects who died within 3 months of ischemic stroke had significantly lower triglyceride levels (median 1.05 mmol/L, IQR 0.65–1.55 mmol/L) compared to survivors (median 1.41 mmol/L, IQR 1.00–2.00 mmol/L; *P* = 0.0035). There were no significant associations between death within 3 months and cholesterol (*P* = 0.1656), HDL (*P* = 0.8137), and LDL (*P* = 0.1797) levels. The association between lipid levels and death within 3 months is given in Table 3. 

### 3.5. Stratified Analysis

 Further, we conducted an independent stratified analysis to test the association between low triglycerides and bad outcome (high mRS) and low triglyceride and death against gender and prior use of statins. This was an exploratory analysis.

 On stratification by gender, we found no significant difference between triglycerides and functional outcome at discharge for males and females (*P* = 0.054 in males versus *P* = 0.17 in females). On stratification by statin, we found a significant difference between triglycerides and functional outcome at discharge for patients who had no history of prior statin use. The analysis revealed that the lower triglycerides were associated with poor outcome at discharge among them (Pearson's correlation coefficient −0.158, 95% CI −0.29 to −0.025; *P* = 0.02). There was no significant association between triglycerides and functional outcome at discharge for patients with prior history of statin use. This is shown in Table 4

On stratification by gender, we found significant association between low triglycerides and death within 3 months for male subjects (*P* = 0.0023) but not for female subjects (*P* = 0.34). On stratification by statin, we found significant association between low triglycerides and death within 3 months for patients with prior history of statin use (*P* = 0.034) and without a prior history of statin use (*P* = 0.0221). This is shown in Table 5.

### 3.6. Logistic Regression and Cox Proportional Hazard Analysis

We analyzed logistic regression model, to adjust for the influences of age and NIHSS on functional outcome at discharge. After adjusting for age, gender, and NIHSS, low triglycerides did not remain an independent predictor of worse functional outcome at discharge (*P* = 0.8046, *R* square =0.493). 

In the multivariate analysis using Cox proportional hazard analysis, only NIHSS remained a significant predictor of 3-month mortality (Hazards ratio 1.12; 95% CI 1.1 to 1.2). The hazard ratios for al the covariates are given in Table 6. However, on removing NIHSS from the model, low triglyceride was significantly associated with mortality at 3 months (Hazards ratio 0.04 for per unit increase in triglyceride, 95% CI 0.001 to 0.64, *P* = 0.02).

## 4. Discussion

Our study revealed that low triglyceride levels were associated with worse stroke as assessed by NIHSS on presentation. This concurs with the results acknowledged by Dziedzic et al., in a study where they correlated the Scandinavian stroke scale in patients to triglyceride levels, measured within 36 hours of arrival [9]. A recent prospective study reported that higher fasting TGs on admission predict less severe disability, reduced disability progression, and all-cause mortality in patients with acute ischemic stroke [15]. Another prospective study confirmed that low serum TG is an independent predictor of mortality after ischemic stroke, but noted that this association did not hold true in the subgroup with cardioembolic stroke [16]. While most studies report a linear association, one group has reported a *J*-shaped curve phenomenon in relation to serum triglycerides and outcome, suggesting that both hypertriglyceridemia and hypotriglyceridemia can be risk factors for poor early outcome in AIS [17]. 

In contrast, a study performed by Simundic et al. found that patients with a higher severity of stroke had higher serum triglycerides; however the relatively small size of their cohort (*n* = 70) may limit the external validity of their results [12]. Lower triglyceride levels have been shown to correlate inversely to stroke infarct volumes on CT scan [18]. Patients who had worse outcomes on discharge from the hospital (mRS ≥ 3) tended to have lower serum triglyceride levels. Li and his associates also similarly reported that triglyceride level was independently associated with poor outcome in their cohort of patients with ischemic stroke [10]. We also found that patients, who died within 3 months, had lower triglyceride levels within the first 24 hours of the stroke than patients who survived. Wier and his associates report similar results in their study of 1310 nondiabetic acute ischemic stroke patients [11]. 

It is known that there are gender differences in lipid levels [19]. Our cohort consisted mostly of older adults. Moreover only 10% of the female population were younger than 50 years, and the average age of menopause reported by the Mayo Clinic for American women is 51 years [20]. It has been reported in the past that on transition from pre- to postmenopausal women often develop features of metabolic syndrome [21] and nearly a 16% increase in triglyceride levels [22]. The analysis of National Health and Nutrition Examination Surveys (NHANES) over 4 decades revealed that the gap in triglyceride levels between men and women narrowed in the 50–59-year age group, and from 60 years onward, women had higher levels than men [23]. Females in our cohort too had higher total cholesterol and HDL levels. For this reason, we performed a gender stratified analysis of the associations between triglycerides and death and found that they remained significant only among male patients. Male patients who died within 3 months of stroke had lower triglycerides, cholesterol, and LDL values. 

On applying regression models to adjust for the effects of age and gender, only triglyceride levels retained significance for predicting death at 3 months. On adding NIHSS to the model, expectedly, this relation no longer held statistical significance. This finding indicates that stroke severity is the main predictor of mortality. However, a low triglyceride level was significantly associated with more severe strokes. 

To explain the association between low triglycerides and worse outcomes after stroke, one common hypothesis put forward by most of the studies is that low triglycerides are surrogate markers of poor prior nutritional status [24] and that this nutritional deficiency leads to a poor outcome after stroke [11, 25, 26]. 

## 5. Limitations 

There are some limitations to our study. As we do not have the data regarding the lipid profiles of patients before the stroke, it is not possible for us to determine whether an acute phase reaction could have affected lipid levels, particularly of triglycerides. Not all patients had lipid levels measured after the stroke. Consequently, the possibility of selection bias cannot be excluded. We also do not have the body mass index or data on other nutritional markers (e.g., prealbumin) of our patients to be able to evaluate the relationship between nutritional status and stroke outcome. 

This is a single center study conducted in an academic medical center with a predominantly white, middle class patient population, and may LIMIT [not limited] applicability to other patient populations.

## 6. Summary

We infer from this hypothesis generating study that a low level of triglycerides predicts poor outcome following stroke and may be used as a prognostic marker for early mortality. 

## Figures and Tables

**Table 1:** Study population characteristics.

Characteristics	(Number of patients) *N* = 334
Age (years)	Median 74 (IQR 62–81.25)
Gender	
Male	185 (55.4%)
Female	149 (44.6%)
Prior treatment with statins	115 (34.4%)
Stroke severity (NIHSS)	Median 4 (IQR 2–9)
Functional outcome at discharge (mRs)	Median 3 (IQR 2–4)
Death within 3 months	32 (9.6%)

**Table 2:** Lipids studies of population at admission with stroke.

Lipids (mmol/L)	Values-median (IQR)
Cholesterol	4.58 (3.82–5.33)
Triglyceride	1.38 (0.96–1.91)
High density lipoprotein (HDL)	1.29 (1.03–1.55)
Low density lipoprotein (LDL)	2.5 (1.91–3.21)

**Table 3:** Lipids values across age, gender, NIHSS, prior statin use, mRS, and death within 3 months.

	Association		*P* value
	Cholesterol (mg/dL)		

Age^#^	Pearson's *ρ* (95% CI)	−0.13 (−0.24 to 0.03)	0.0149∗
Gender			
Males	Median (IQR)	4.45 (3.62 to 5.26)	0.0037∗
Females	Median (IQR)	4.72 (4.1 to 5.4)	
NIHSS^#^	Pearson's *ρ* (95% CI)	0.032 (−0.08 to 0.14)	0.572
Statins			
Yes	Median (IQR)	4.03 (3.44 to 5.02)	<0.0001∗
No	Median (IQR)	4.78 (4.06 to 5.46)	
mRs	Pearson's *ρ* (95% CI)	−0.056 (−0.163 to 0.052)	0.31
Death within 3 months			
Yes	Median (IQR)	4.23 (3.16 to 4.88)	0.166
No	Median (IQR)	4.58 (3.87 to 5.33)	

	Triglycerides^#^ (mg/dL)		

Age^#^	Pearson's *ρ* (95% CI)	−0.12 (−0.22 to 0.01)	0.034∗
Gender			
Males	Median (IQR)	1.37 (0.95 to 1.84)	0.6992
Females	Median (IQR)	1.41 (0.97 to 2.02)	
NIHSS^#^	Pearson's *ρ* (95% CI)	−0.16 (−0.27 to −0.05)	0.004∗
Statins			
Yes	Median (IQR)	1.51 (1.09 to 2.21)	0.0056∗
No	Median (IQR)	1.28 (0.91 to 1.76)	
mRs	Pearson's *ρ* (95% CI)	−0.128 (−0.232 to −0.021)	0.02∗
Death within 3 months			
Yes	Median (IQR)	1.05 (0.65 to 1.55)	0.0035∗
No	Median (IQR)	1.41 (1.00 to 2.01)	

	HDL (mg/dL)		

Age	Pearson's *ρ* (95% CI)	0.09 (−0.02 to 0.196)	0.1
Gender			
Males	Median (IQR)	1.16 (0.98 to 1.42)	<0.0001∗
Females	Median (IQR)	1.4 (1.18 to 1.69)	
NIHSS	Pearson's *ρ* (95% CI)	0.026 (−0.08 to 0.14)	0.64
Statins			
Yes	Median (IQR)	1.16 (0.96 to 1.4)	0.0016∗
No	Median (IQR)	1.34 (1.11 to 1.63)	
mRs	Pearson's *ρ* (95% CI)	0.034 (−0.074 to 0.141)	0.54
Death within 3 months			
Yes	Median (IQR)	1.2 (0.97 to 1.73)	0.8137
No	Median (IQR)	1.29 (1.03 to 1.53)	

	LDL^#^ (mg/dL)		

Age^#^	Pearson's *ρ* (95% CI)	−0.16 (−0.26 to 0.05)	0.005∗
Gender			
Males	Median (IQR)	2.33 (1.76 to 3.19)	0.0526
Females	Median (IQR)	2.65 (2.1 to 3.21)	
NIHSS^#^	Pearson's *ρ* (95% CI)	0.067 (−0.04 to 0.18)	0.238
Statins			
Yes	Median (IQR)	2.1 (1.68 to 2.65)	<0.0001∗
No	Median (IQR)	2.72 (2.1 to 3.39)	
mRs	Pearson's *ρ* (95% CI)	−0.056 (−0.163 to 0.053)	0.32
Death within 3 months			
Yes	Median (IQR)	2.41 (1.63 to 2.97)	0.1797
No	Median (IQR)	2.53 (1.98 to 3.22)	

^#^Log of variables was used to calculate the association due to their non-normal distribution.

**Table 4:** Association between triglyceride^#^ and mRs stratified by statin use and gender.

	Pearson's correlation coefficient	95% CI	*P* value
Triglyceride (with no prior statin use)	−0.16	−0.29 to −0.03	0.02∗
Triglyceride (with prior statin use)	−0.05	−0.23 to 0.13	0.59
Triglycerides in males	−0.14	−0.28 to 0.003	0.05
Triglycerides in females	−0.11	−0.27 to 0.05	0.17

^#^Log of triglyceride was used to test the associations as it had a nonnormal distribution.

**Table 5:** Association between triglyceride^#^ and death stratified by statin use and gender.

	Death ≤ 3 months	Alive at 3 months	*P* value
Triglyceride (with no prior statin use)	1.00 (0.67 to 1.47)	1.31 (0.93 to 1.78)	0.0345∗
Triglyceride (with prior statin use)	1.24 (0.63 to 1.78)	1.55 (1.15 to 2.28)	0.0221∗
Triglycerides in males	1.00 (0.63 to 1.31)	1.39 (1.00 to 1.94)	0.0023∗
Triglycerides in females	1.29 (0.86 to 1.75)	1.41 (1.00 to 2.03)	0.3405

^#^Log of triglyceride was used to test the associations as it had a nonnormal distribution.

**Table 6:** Cox regression analysis for all patients: overall *χ* ^2^ ≤ 0.0001.

Variable	Covariate	Coeff (*b*)	SE (*b*)	*P*	Hazards ratio	95% CI
	Triglyceride	−0.004	0.004	0.26	0.99	0.99–1.0
Mortality at 3 months	NIHSS	0.11	0.02	<0.001∗	1.12	1.1–1.2
	Age	−0.01	0.01	0.28	0.99	0.97–1.0
	Female gender	−0.01	0.19	0.97	0.99	0.5–2.1
